# What is behind the lifestyle risk factors for head and neck cancer?

**DOI:** 10.3389/fpsyg.2022.960638

**Published:** 2022-10-13

**Authors:** Anem Iftikhar, Mohammad Islam, Simon Shepherd, Sarah Jones, Ian Ellis

**Affiliations:** ^1^Unit of Cell and Molecular Biology, Dundee Dental School, University of Dundee, Dundee,United Kingdom; ^2^Department of Oral Surgery and Medicine, Dundee Dental Hospital, Dundee, Scotland, United Kingdom

**Keywords:** cancer, stress, alcohol, smoking, glucocorticoids, HPA-axis, lifestyle

## Abstract

Lifestyle factors are known to be influential determinants of health. According to the World Health Organization (WHO), approximately one third of deaths involve unhealthy lifestyle habits. Among lifestyle risk factors for head and neck cancers (HNC), alcohol consumption and smoking have an undeniable role in the multifactorial aetiology of the disease. In recent years, the promotion of healthy lifestyle choices has gained significant attention as contributory to improving health and disease prevention. Interventions to tackle these risk factors are vitally important in disease prevention and progression. However, in order to effectively prevent the disease and reduce the risk factors, it is crucial to identify what upstream reasons lead to the adoption of these lifestyle risk factors in the first place. Stress being a constant aspect of modern-day life is known to contribute to alcohol and smoking practices. In this review paper, relevant literature was searched in PubMed database for stress, lifestyle factors, HNC and cancer to explore the role of stress and its associated biological pathways as an upstream factor in the adoption of lifestyle risk factors that cause HNC. It highlights the importance of stress pathways and the Hypothalamus Pituitary Adrenal (HPA) axis as a locus of interaction between stress, alcohol, smoking and cancer. Despite their widely accepted harmful effects, alcohol and smoking remain deeply rooted in contemporary life. A greater understanding of the impact of stress on lifestyle choices and an exploration of the mechanisms resulting in stress, alcohol- and smoking- related cancer may highlight opportunities for improved prevention measures through the modification of unhealthy lifestyle choices.

## Introduction

“There comes a point where we need to stop just pulling people out of the river, we need to go upstream and find out why they are falling in.” Desmond Tutu.

To live a life to one’s full potential, good health is a key element. Strongly intertwined with health and disease, is the Selye’s concept of stress, described as an occurrence when environmental demands are beyond one’s perspective of the ability to cope. Whereas acute stress enhances the body’s defense ability, long-term stress impacts behavior, well-being, and imposes health burden (Viner, 1999).

In addition to the biochemical pathways through which stress affects health and brings illness, it also impacts the lifestyle choices, and these lifestyle factors form an important component of a disease aetiology. HNC has a recognized lifestyle aetiology in which alcohol and smoking are independent as well as synergistic risk factors for cancer initiation and progression.

The intention of this review is to highlight the role of stress as an upstream factor in the well-recognized lifestyle risk aetiology of HNC. Relevant literature was searched in PubMed database for health, stress, lifestyle factors and HNC to explore the role of stress and its associated biological pathways as an upstream factor in the adoption of lifestyle risk factors that cause HNC. One of the limitations was existence of extensive literature in these areas separately, for example stress and alcohol, stress and smoking, HNC and lifestyle risk factors but lack of studies combining stress, lifestyle risk factors and HNC. This proceeding section briefly outlines health, lifestyle risk factors and HNC, followed by the journey of alcohol and smoking from lifestyle to lifestyle risk factors, alcohol and smoking in HNC, the stress factor behind the lifestyle risk factors aetiology, the underlying mechanisms that connect the stressful events to adoption and continuation of lifestyle risk factors and the intersection of stress, lifestyle factors and cancer at the hypothalamus pituitary adrenal (HPA) axis.

### Health, lifestyle risk factors, and HNC

The concept and definition of health has evolved with the growth and evolution in scientific knowledge. In the early 19th and 20th centuries, health was largely defined by the absence of disease in the physical condition. The WHO, since 1948, has included in the definition of health as being “*a state of complete physical, mental and social well-being, not merely the absence of infirmity or disease.” The first International Conference on Health promotion, held in Ottawa in 1986, led to an expansion to this definition, “To reach a state of complete physical, mental, and social well-being, an individual or group must be able to identify and to realize aspirations, to satisfy needs, and to change or cope with the environment. Health is, therefore, seen as a resource for everyday life, not the objective of living. Health is a positive concept emphasizing social and personal resources, as well as physical capacities. Therefore, health promotion is not just the responsibility of the health sector but goes beyond healthy life-styles to well-being* (World Health Organization, 1986).”

At that time, this expanded definition was welcomed as health was released from the confines of just physical condition and rather included mental and social constraints of health. With the rise in life expectancy and individuals living with a greater burden of chronic conditions this definition has become ever more problematic since the attainment of such a complete state of health is unachievable. As such, newer definitions of health have been promoted (Leonardi, 2018; Fallon and Karlawish, 2019). Last’s Dictionary of Public Health further defines health as “A structural, functional and emotional state that is compatible with effective life as an individual and as a member of family and community groups” (Last, 2007).

As the definition of health has evolved, so have the factors that determine it and these are known as the determinants of health. Broadly, they can be divided into the four main categories of nutrition, lifestyle, genetic and environmental. Political, economic, social, and cultural factors also influence one’s health and are categorized as the wider determinants of health (World Health Organization, 1986; Public Health England, 2018).

Whereas genetic and biological factors are important in the transition from health to disease, lifestyle risk factors have gained significant attention in recent years. Numerous disease processes are known to be significantly influenced by these lifestyle factors. HNC is understood to be of a multifactorial casation. Various factors are involved in the initiation, progression, and metastasis of the tumor. The overall aetiology and risk factors of HNC have been classified in different ways (Kumar et al., 2016; Irani, 2020; Aghiorghiesei et al., 2022) and are briefly summarized in Table 1. These tumors are certainly recognized as having important and modifiable lifestyle risk factor aetiology (Zaman et al., 2019). Alcohol consumption, tobacco usage, betel quid and dietary deficiency are the most important lifestyle risk factors for HNC (Petti, 2009).

**Table 1:** Summarizing the risk factors related to head and neck cancer (HNC).

Risk factors	References
*Demographics*	
Age	Conway et al., 2010; Curtis et al., 2020; Park et al., 2022
Ethnicity	
Socio-economic Status	
*Lifestyle*	
Smoking	Goldenberg 2002; Petti 2009
Alcohol consumption	
Betel quid/ Areca nut/ Pan/ Tobacco chewing	
Diet	
Physical inactivity	
Mate drinking	
*Infections and Immunosuppression*	
HPV	Andy et al., 2003; Mazul et al., 2020; Sabatini and Chiocca 2020
HIV	
*Genetic alterations*	
Errors in DNA replication machinery	Ali et al., 2017; Huang et al., 2019
Altered signaling pathways	
Chromosomal instability	
Changes in TSG and oncogenes	
*Environmental factors*	
UV radiation	Su et al., 2010; Agrawal et al., 2013
Heavy metals	
*Oral potentially malignant disorders*	
Leukoplakia	Speight et al., 2018
Erythroplakia	

The rising global incidence of HNC has attracted much attention and interventions to modify these risk factors. These interventions are important not only to the clinicians charged with implementing them, but also to the patients who need to know what changes they can make to positively influence disease progression following diagnosis (Zaman et al., 2019). According to WHO, an estimated of one third of deaths from cancer are due to tobacco use, high body mass index, alcohol use, low fruit and vegetable intake, and lack of physical activity (International Agency for Research on Cancer, 2020).

Therefore, health improvement or disease prevention requires an understanding of the lifestyle choices that eventually become the risk factors in the onset or progression of the disease and more importantly, the upstream reasons for these choices. Thirty to Fifty percent of all cancer deaths could be prevented by adopting healthier lifestyles (World Health Organization, 2022). Recent reviews have examined the direct and indirect role (Iftikhar et al., 2021a,b) that stress hormones play in the progression of HNC cancer. This current review explores the importance of stress as an upstream reason for the initiation and continuation of tobacco and alcohol use. It also looks into the biological mechanisms by which stressful events and the resultant dysregulated stress pathways play a role in the adoption and continuation of lifestyle risk factors.

### Alcohol and smoking–from lifestyle to lifestyle risk factors: An overview of the historical journey

Lifestyle is a broad concept. In simple words, it is usually described as the way people live. It spans across different levels and is governed not only by individual choices, but reflects geographical, economic, political, cultural, and religious practices. At an individual level, it is determined by everyday repeated behaviors and activities (Al-Maskari, 2010).

Alcohol consumption and smoking slowly became deeply rooted into lifestyle at a cultural, societal, and at an individual level. These firmly embedded lifestyle habits often cannot be attributed to a single cause, as they serve multiple purposes such as facilitating social interactions, signifying joy and festivity, offering a means to relax, as an escape from hurt and as a mechanism to cope with stressful periods (Mann et al., 2000). Despite knowing the associated harmful effects, people continue to smoke and drink alcohol due to the pleasure associated with them or the crutch they provide.

The first reported use of tobacco is when Central Americans started using tobacco leaves as part of social and religious ceremonies (Mishra and Mishra, 2013). This practice slowly spread, and it grew for spiritual as well as medicinal purposes. Tobacco use was seen as a cure for toothache, earache, headache and depression. By the 14th century tobacco was considered a powerful remedy and widely seen as a universal cure. Later, in the 15th and 16th centuries, it reached Europe where it developed a similar reputation of a curative for a vast range of disorders, a relaxant and that of a holy herb (Mullett, 1940; Stewart, 1967). Children were told to smoke tobacco in school rooms during the London plague of 1,665 to prevent the harmful effects of bad air (Stewart, 1967). By the 17th century tobacco use had spread around China, India, Middle East, Japan, South-East Asia, and West Africa. Various forms were snuffed, chewed, drunk, yet smoking remained dominant and enjoyed during gatherings in the form of cigars, pipes, and hookahs (Stewart, 1967).

Tobacco and alcohol were further triumphant during times of war. Cigar usage became popular amongst military men in the Peninsular war. Cigarettes were said to “lighten the hardships of war” and bring “solace of the wounded” during World War I (Smith and Malone, 2009). The war difficulties for soldiers were compensated for through regular rations of rum and cigarettes. Alcohol and cigarettes held a position of morale boosting and motivational psychological effects and were therefore seen more of a necessity than a luxury. The war further paved the way for cigarette smoking as an acceptable behavior (Reeve, 2016).

However, even during the era where tobacco gained popularity for its medicinal properties, there was dispute and questioning of its widely believed efficacy (Mullett, 1940), and its ability to decrease the body’s resistance to disease (The Germicidal Properties of Tobacco Smoke, 1913). In a counter-attack on tobacco, King James I of England wrote, “a custom loathsome to the eye, hateful to the nose, harmful to the brain, dangerous to the lungs, and in the black, stinking fume thereof, nearest resembling the horrible Stygian smoke of the pit that is bottomless (Milne, 2011).” Studies associating lung cancer to smoking started emerging in the 1920’s with reports establishing a causal link in 1950’s; however the reaction from the medical community and tobacco industry was delayed (Proctor, 2004).

The earliest evidence for alcoholic drinks comes from China in 7000 BC, as a fortuitous finding from grapes and berries left in clay vessels producing a fermented drink. The purposeful cultivation of plants for alcohol production happened in Fertile Crescent–modern day regions of Lebanon, Iraq, Israel, Syria, Jordan, and Northern Egypt. Inhabitants of Iran used wine as a method to store grapes and they benefited from its nutritional value (Mann et al., 2000). Soon, in many cultures, alcohol started serving purposes wider than just a nutritional staple. It was greatly valued by early Sumerians, Egyptians, Greeks, and Romans (4,000–2,000 BC). It signified celebration, spirituality and eliteness (Khaderi, 2019).

In early Egyptian culture, commoners drank beer with wine reserved as a drink for rulers. It was viewed as a currency to exchange goods between countries, as a medicine for wound healing and cancer, as an analgesic, as a relaxant, and as a means of pleasure. It was the Greeks who were the first to identify its harmful aspects, in addition to its benefits. In all of these cultures it was viewed as a religious fulfillment (Mann et al., 2000). Alcohol played an important role in the religious duties of Jews and Christians with recommendations to abstain for those who could not control their drinking habit. Islam (600 AD) completely forbade its followers from alcohol indulgence. The Muslim surgeon Al Zahwari identified that alcohol caused convulsions, dementia, paralysis and liver problems in his patients (Khaderi, 2019).

Alcohol-associated problems increased when distillation became more common. Distilled products contain 30% more alcohol. Distillation was first established around the year 800 AD and flourished in Germany by the 15th Century. Distilled alcohols were sold as a health tonic with benefits ranging from a cold cure to comforting the heart, which spread to America and Europe shortly thereafter (Smith and Malone, 2009). Gradually, drunkenness became a widespread issue. Britain experienced a peak in alcohol consumption by mid-18th century, a period known as the gin craze. A rise in social, domestic and criminal issues as well as medical complications was associated with gin, as communicated through the Gin Lane, a classic imagery by William Hogarth, detailing the physical and mental decline of the community. Hogarth’s Gin Lane supported the Gin Act of 1751, which greatly reduced gin’s consumption (Harris, 2011). A century later, a similar escalation in alcohol consumption was seen in the US and temperance groups started demanding prohibition, which was legally enforced in 1919–1933. Prohibition led to massive economic and job losses, and it was subsequently repealed (Mann et al., 2000).

Alcohol consumption and smoking practices still remain widespread across many cultures. Factors such as easy availability, social acceptance, heavy advertisement and economic prosperity contribute to their high prevalence. The far-reaching harmful effects along with the associated burden of chronic diseases, attributable to smoking and alcohol, has led to an increase in research efforts, awareness raising methods and the implementation of prevention strategies.

### Alcohol and smoking in HNC

Every year an estimated half a million HNC cases are reported worldwide. Smoking and alcohol consumption are the two major risk factors and epidemiological studies worldwide report their association with HNC. A number of studies significantly report their independent and joint synergistic association to HNC (Warnakulasuriya, 2009; Mello et al., 2019).

Tobacco products have been an established cause of cancer for many decades. According to the WHO global report on trends of tobacco use (2000–2025), around 1.3 billion people worldwide are tobacco users (World Health Organization, 2021) leading to an estimated 2.4 million tobacco and smoking related-cancer deaths occurring worldwide annually (Irani, 2020). The International Agency for the Research on Cancer (IARC) Monographs (I) associate tobacco with 20 types of cancers, including HNC and aerodigestive tract IARC (IARC Working Group on the Evaluation of Carcinogenic Risks to Humans, 2004, 2012). The causal relationship of smoking to lip cancer was first established in 1964 and to other oropharyngeal cancers in 1971 (National Center for Chronic Disease Prevention and Health Promotion Office on Smoking and Health, 2014). Many subsequent studies have found an aetiological link between alcohol, smoking and oral cancer (Moreno-López et al., 2000; Lu et al., 2018; Porter et al., 2018; Chang et al., 2020).

Alcohol consumption has been consistently linked to HNC. The first published study on the association of alcohol with cancer mortality was in 1903 and many studies have supported this since (Martinez, 1969; Olsen et al., 1985; Bagnardi et al., 2015). In 1988, IARC classified alcohol as a carcinogen for oral, pharyngeal, laryngeal, esophageal and liver cancers. A large body of epidemiological data over many years support the strong association of alcohol with oral and pharyngeal cancers (Wynder and Bross, 1957; Goldstein et al., 2010). A meta-analysis reported that in addition to moderate and heavy drinking, light drinking also increases the risk of oral, pharyngeal and esophageal cancers (Choi et al., 2018). In 2020, these cancers contributed to 562,328 cases and 277,597 deaths globally (IARC, 2020).

In 2012, 5.5% of all cancer cases and 5.8% of cancer deaths were attributable to alcohol (Praud et al., 2016; LoConte et al., 2018). In 2016 4.8% and in 2019 4.9% of all cancer deaths were attributable to alcohol (GBD 2019 Risk Factors Collaborators, 2020; Shield et al., 2020). In 2020, a small decrease was noted and an estimated 4% of all cancer cases were attributable to alcohol. Cancers with the highest population attributable fractions (PAF) were of the oesophagus (31.6%), pharynx (22.0%), and lip and oral cavity (20.2%). Seventy-seven percent of all alcohol-attributable cases were found in men and 23% in women (GBD 2019 Risk Factors Collaborators, 2020). Studies have also reported significantly greater odds of oral squamous cell carcinoma as a result of synergistic consumption of alcohol and smoking (Mello et al., 2019).

### The stress factor behind the lifestyle risk factors

As early as smoking and alcohol were discovered, one of the reasons for their consumption was to escape from hurt, negative feelings, and difficult times (Mann et al., 2000; Ritz and Orth, 2000). Stress can be described as any condition, adverse environment, experience, or perceived threat that challenges the coping ability of an individual. Stressful events can generate reactions and result in adoption of unhealthy lifestyle behaviors. Therefore, it is important to identify how much of a contributory role stress does play in the adoption of lifestyle risk factors in order to effectively eradicate them and prevent associated diseases.

Some studies argue that smoking may not be a lifestyle, but a helpless response to social and economic conditions (Graham et al., 2006). A number of studies in contemporary literature have reported anxiety, emotional problems, and stress as motives for individuals to regularly smoke cigarettes (Lerman et al., 1996; Lawn et al., 2002; Thompson et al., 2003; Kerr et al., 2006; McEwen et al., 2008; Fidler and West, 2009; Clancy et al., 2013).

Higher perceived stress levels are associated with greater odds of smoking (Gallo et al., 2014), with perceived stress in smokers and ex-smokers strongly linked to smoking (Tsourtos et al., 2011) and positively related to nicotine withdrawal symptoms (Lawless et al., 2015). As such stress, in part, may be responsible for perpetuating smoking habits (Bryant et al., 2011; Slopen et al., 2013). Lasser et al. reported that people with mental illness were twice as likely to smoke than people without mental illness (Lasser et al., 2000). Tobia et al. reported anxiety disorders as an important risk factor for tobacco consumption. It has been estimated that approximately 33% of all tobacco is consumed by people who live with some form of mental disorder for a duration of 12 months, of which anxiety accounted for a half (Tobias et al., 2008). Research exploring the tobacco industry documentation suggests that the psychologically vulnerable, new starters and smokers concerned about health were targeted *via* positive and independent lifestyle imagery, increased masculinity, and independently facing solitude without loneliness (Lasser et al., 2000; Pollay, 2000).

Studies suggest that individuals with anxiety may smoke to alleviate symptoms. However, reports of the opposite direction of causality where smoking may result in higher levels of stress, but not vice versa, also exist (Hajek et al., 2010). Todd et al. observed changes in acute stress levels between heavy, light and non-smokers. The induction of stress caused heavy smokers a greater urge to smoke and they expressed more stress in comparison with light and non-smokers (Carim-Todd et al., 2016). A study examining the temporal relationship of antecedent smoking to later onset of depressed mood, found increased association of smoking with subsequent depressed mood (Wu and Anthony, 1999).

An association between smoking and mental illness has been well recognized; however, the direction of the association has been clouded by the complexity, and existence of mixed or inconsistent evidence (Moylan et al., 2012; Garey et al., 2020). A recent systematic review reported evidence for baseline smoking leading to mental illness and vice versa, with fewer studies showing a bidirectional relationship (Fluharty et al., 2017).

Several studies have demonstrated the association of alcohol related outcomes such as alcohol misuse, alcoholism and heavy drinking in response to stressful life experiences, perceived stress and chronic stressors (Cole et al., 1990; Wilsnack et al., 1991). There is a causal relationship between stress and alcohol consumption (Russell et al., 1999).

Experimental stress induction leads to increased alcohol consumption (Amlung and Mac, 2014), craving (Fox et al., 2007), and relapse (Noone et al., 1999), in individuals with alcohol use disorder (AUD), and in heavy social drinkers (Field and Quigley, 2009). People with alcohol dependence but not seeking treatment, showed increased consumption following acute psychosocial stressors (Thomas et al., 2011). Wit et al., reported that acute stress produced a modest increase in alcohol consumption even in healthy social drinkers (de Wit et al., 2003), while Magrys and Olmstead reported increased voluntary alcohol intake amongst undergraduate students in response to acute stress (Magrys and Olmstead, 2015). Individuals with feelings of increased stress, anxiety, hopelessness, and fear were more likely to increase their drinking during the COVID-19 pandemic (Lechner et al., 2020; Thompson et al., 2021).

Epidemiological studies also report a positive correlation between smoking and alcohol use. Tobacco smokers tend to misuse alcohol more than non-smokers. This combination of alcohol and smoking is more likely to happen in adolescence (Cross et al., 2017).

The evidence linking a temporal stressful exposure with subsequent unhealthy patterns of tobacco smoking and alcohol consumption, and the association of these factors with the aetiology of HNC necessitates a greater understanding of the underlying mechanisms that link stress, lifestyle factors and cancer.

### The intersection of stress, alcohol, smoking, cancer, and the HPA axis

Research reveals the existence of complex interactions between environmental conditions and individual predisposition to alcohol consumption and smoking. Epidemiological studies suggest stress can trigger pathological alcohol consumption and smoking (Cross et al., 2017). Exposure to stress occurs in multiple aspects of life. The type of stressors include demanding work environments, family stresses (divorce, break up, sickness, or death of a family member), low income, childhood maltreatment. The biological systems involved in the stress response are the HPA axis and autonomic nervous system (ANS), which release glucocorticoids and catecholamines (Doyon et al., 2013). This section looks at the underlying biological mechanisms that connect prior stressful experiences to adoption of unhealthy lifestyle risk factors (alcohol and smoking) for cancer.

Any stressful situation is recognized by the central nervous system (CNS) which activates the HPA axis and sympathetic nervous system (SNS). The hypothalamus releases corticotropin-releasing hormone (CRH) that causes the pituitary gland to produce adrenocorticotropic hormone (ACTH). ACTH results in the release of glucocorticoids from the adrenal cortex, mainly cortisone. This undergoes enzymatic conversion to the active form, cortisol, in the target organs, shaping the stress response and helping the body survive any stressful event and regain homeostasis (Iftikhar et al., 2021a).

Prolonged exposure to stress can alter the dynamics of the HPA axis and increase the cortisol burden. Childhood or neonatal exposure to stressful events and adversity may result in genetic modification of the glucocorticoid genes affecting glucocorticoid release and stress responsivity (Keyes et al., 2012). This may link childhood adversity as a risk factor for the development of anxiety disorders in adulthood and contribute to vulnerability to alcoholism in later life. Other stress genes are also reported to interact with environmental factors to increase the risk of AUD (Stephens and Wand, 2012).

The altered activity of the HPA axis as a result of stressful events present prior to alcohol use may act as a risk factor and increase the vulnerability to alcohol disorder. Alcohol initially may act as an anxiolytic, reducing anxiety yet simultaneously acting as a stressor by activating the HPA axis and increasing the release of glucocorticoids, an increase which is also observed in social drinkers (Waltman et al., 1993).

The dysregulated HPA axis and elevated levels of cortisol also play a role in the dependence and rewarding effect of alcohol. Alcohol, smoking or any other drug dependence includes behavior reinforcement, which involves the mesolimbic dopamine system. The mesolimbic dopamine system is a locus of interaction for the synergistic activities of alcohol, nicotine and stress. Alcohol and other drugs can increase the dopaminergic activity which include increased dopamine firing into the nucleus accumbens. This causes an excitatory or rewarding effect of the drug (Boileau et al., 2003). Whereas acute alcohol intoxication may result in increased dopaminergic release, chronic alcohol use may cause lower than normal dopamine release which might prompt alcohol seeking behavior to maintain the same effect (Okamoto et al., 2006). HPA pathway activation by chronic alcohol consumption releases glucocorticoids, which increase the motivational properties of alcohol by increasing dopamine release, as well as inhibiting its clearance, and potentiating alcohol’s effect. Thus, chronic alcohol intake may increase cortisol levels, which in turn play a role in drug reinforcement by mediating the dopaminergic reward pathway and playing a role in maintenance of alcohol consumption (Vendruscolo et al., 2012). The secretion of cortisol and norepinephrine in response to stress is involved in habit-based memory consolidation, which is involved in alcohol dependence (Stephens and Wand, 2012).

Like alcohol, chronic smoking is also associated with altered functioning of stress pathways. Acute nicotine administration activates the HPA and causes a resultant increase in cortisol. Nicotine is reported to activate the HPA axis in a dose dependent manner. Higher basal cortisol levels and stressful life events are associated with increased smoking initiation and maintenance (Rao et al., 2009). Increased basal cortisol was reported in habitual smokers throughout the day (Kirschbaum et al., 1994). Whereas basal cortisol levels were elevated in habitual smokers, blunted cortisol levels were reported in response to stress when compared to non-smokers (Kirschbaum et al., 1993; Childs and de Wit, 2009). Combined psychological stress and smoking have an additive effect on cortisol release compared to a stressful task or cigarette alone (Pomerleau and Pomerleau, 1990). Like alcohol, stress and nicotine may have additive effects on cortisol levels which may in turn reinforce smoking intensity and result in smoking maintenance (McKee et al., 2011).

HPA activity during abstinence from habitual smoking showed elevated resting cortisol and blunted levels in response to stress; however, there are only limited studies to confirm this (Al'Absi et al., 2005). Studies show that HPA and resultant cortisol release is dysregulated in smokers. Findings from various studies imply that HPA axis dysregulation, in response to stressful events, is a risk for smoking onset. However, studies also suggest that increased cortisol, as a result of a dysregulated HPA axis, plays a stronger role in sustained smoking and difficulty in cessation, than initiation (Raffetti et al., 2021).

Alcohol and smoking are known lifestyle risk factors for HNC. Alcoholism is ten times more prevalent in smokers than in non-smokers. Their synergistic presence increases the odds of HNC significantly. Alcohol consumption alone leads to a nine-fold increased risk of developing HNC (Olshan et al., 2001), smoking alone brings a 10-fold increase, however, the synergistic effect of smoking and alcohol in combination leads to a 35-fold increased risk (Dal Maso et al., 2016). Interestingly, stress also plays a role in this synergism. Studies have shown the role of glucocorticoids in increased alcohol self-administration, secondary to nicotine by potentiating the dopaminergic activity in the mesolimbic dopamine system (Doyon et al., 2013).

**Figure 1:**
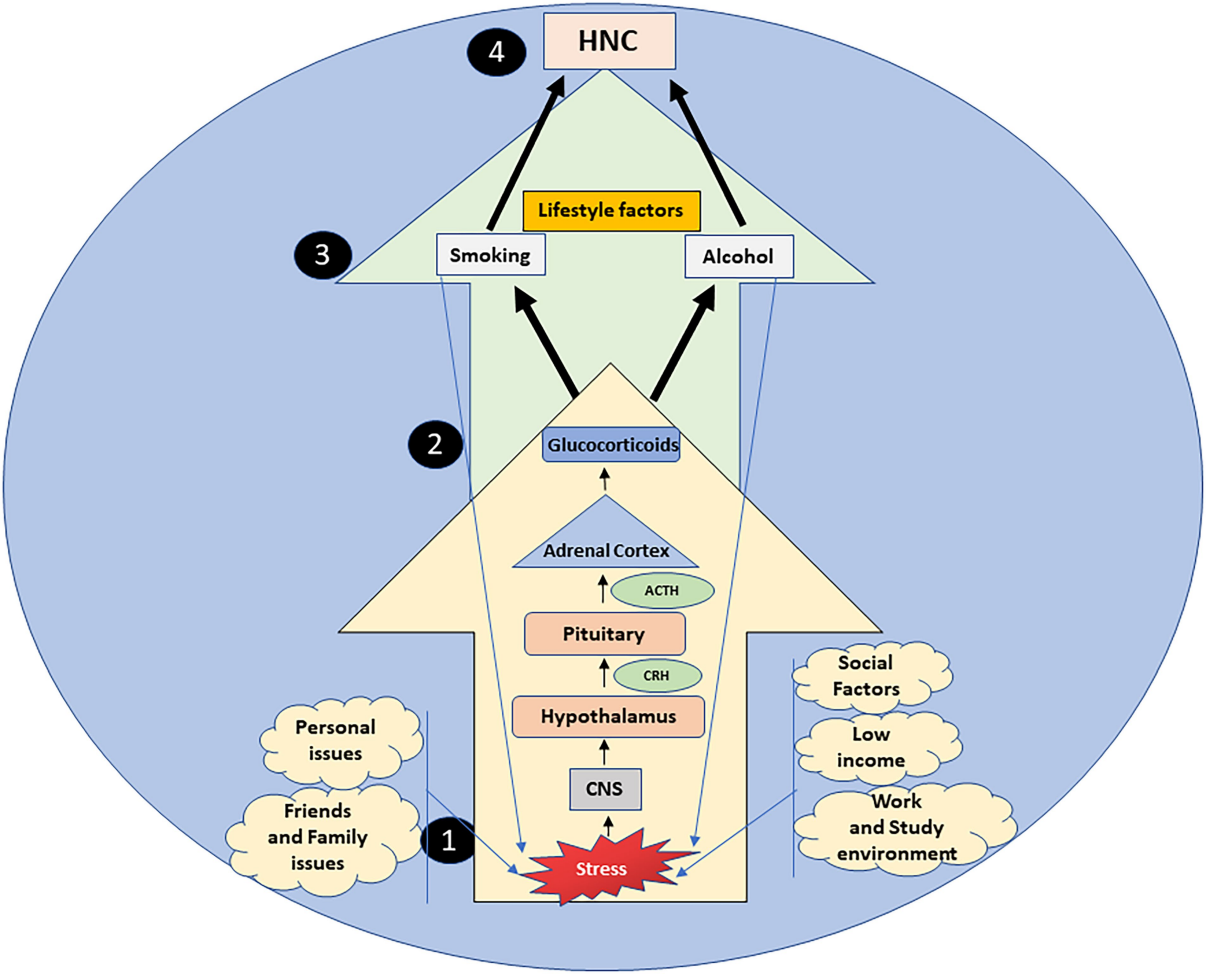
Stress, a constant aspect of everyday life, is a serious upstream reason in the adoption and continuation of alcohol consumption and smoking via the HPA axis. Stressful situations arising from multiple stressors (1), are processed by the central nervous system (CNS), that activates the hypothalamus pituitary adrenal (HPA) axis (2). The hypothalamus, pituitary gland and adrenal cortex cause the sequential release of corticotropin-releasing hormone (CRH), adrenocorticotropin releasing hormone (ACTH) and glucocorticoids respectively (2). Prolonged exposure to stress can increase the burden of glucocorticoids, enhancing the vulnerability to lifestyle risk factors (alcohol and smoking) (3), that initially act as anxiolytics, yet simultaneously as stressors, increasing the HPA axis activity. Thus, stress through the deregulated HPA axis acts as an upstream factor in the lifestyle risk etiology of head and neck cancers (HNC) (4).

The evidence referenced in this paper shows that stress, through the HPA axis and the sequential release of CRH, ACTH, and glucocorticoids, also acts as an upstream reason for adoption and maintenance of lifestyle risk factors that cause HNC, thus signifying the importance of stress pathways and the HPA axis as a locus of interaction for stress, alcohol, smoking, and cancer (Figure 1). This could also signify that lifestyle factors are not only a risk factor due to their well-known carcinogenic mechanisms (Ogden, 2018), but also through their ability to keep stress pathways activated, that in turn aid tumor progression directly by affecting cell proliferation, DNA damage and signaling pathways, and also increase the odds of adopting risky lifestyle behaviors *via* underlying biological mechanisms (Boileau et al., 2003; Okamoto et al., 2006).

## Conclusion

Stress is any condition, adverse environment, experience or perceived threat that challenges the coping ability of an individual. Stressful events influence lifestyle risk factors such that stressors affect their onset and continuation (Viner, 1999). However, the onset of risk factors is a complex interaction between environmental, genetics and individual factors (Petti, 2009). Studies (Keyes et al., 2012; Stephens and Wand, 2012) show that childhood adversity and stressful prenatal events can result in gene modifications making an individual more prone to anxiety states, a dysregulated HPA axis and adoption of risky lifestyle behaviors later in life.

Cultural factors, ease of availability and social acceptance also impact lifestyle choices. A very important consideration is that it is not merely the stressor, but also the stress response of an individual that determines the effect of the stressor. The same level of stress may feel different for individuals and thus may not provoke the same reactions increasing the challenges of stress research (Zaman et al., 2019).

A burgeoning mental health crisis has certainly been exacerbated by the recent global COVID-19 pandemic. Mental health impacts the progression of HNC directly and indirectly through lifestyle choices and therefore efforts should be made to go upstream and address the reasons that lead to unhealthy lifestyle choices at their origins. The combined efforts of research, government and science will be needed to explore deeply the intersection of stress, lifestyle and disease in order to generate effective public health measures to mitigate stress and combat unhealthy lifestyle measures that constitute the etiology of HNC.

## Author contributions

AI, MI, SS, SJ, and IE: conceptualisation. AI: methodology. AI and MI: software and investigation. AI, SJ, and IE: writing—original draft preparation. AI, MI, SS, SJ, and IE: writing—review and editing. IE, SJ, and SS: supervision. AI, IE, and SJ: project administration. SJ and IE: funding acquisition. All authors contributed to the article and approved the submitted version.

## Funding

This research was funded by University of Dundee Global Challenges Research Funding Scholarship Awarded to AI under supervision of IE and SJ.

## Conflict of interest

The authors declare that the research was conducted in the absence of any commercial or financial relationships that could be construed as a potential conflict of interest.

## Publisher’s note

All claims expressed in this article are solely those of the authors and do not necessarily represent those of their affiliated organizations, or those of the publisher, the editors and the reviewers. Any product that may be evaluated in this article, or claim that may be made by its manufacturer, is not guaranteed or endorsed by the publisher.

## Abbreviations

HNC: Head and neck cancer
HPA: Hypothalamus pituitary adrenal axis
IARC: International Agency of Research on Cancer
AUD: Alcohol use disorder
WHO: World Health Organization
CRH: Corticotropin releasing hormone
ACTH: Adrenocorticotropic hormone

## References

[ref1] Aghiorghiesei O. Zanoaga O. Nutu A. Braicu C. Campian R. S. Lucaciu O.. (2022). The world of Oral cancer and its risk factors viewed from the aspect of MicroRNA expression patterns. Genes 13:594. doi: 10.3390/genes13040594, PMID: 35456400 PMC9027895

[ref2] Agrawal A. Shindell E. Jordan F. Baeva L. Pfefer J. Godar D. E. (2013). UV radiation increases carcinogenic risks for oral tissues compared to skin. Photochem. Photobiol. 89, 1193–1198. doi: 10.1111/php.12140 23855371

[ref3] Al'Absi M. Hatsukami D. Davis G. L. (2005). Attenuated adrenocorticotropic responses to psychological stress are associated with early Smoking relapse. Psychopharmacology 181, 107–117. doi: 10.1007/s00213-005-2225-3, PMID: 15834539

[ref4] Ali J. Sabiha B. Jan H. U. Haider S. A. Khan A. A. Ali S. S. (2017). Genetic etiology of oral cancer. Oral Oncol. 70, 23–28. doi: 10.1016/j.oraloncology.2017.05.004 28622887

[ref5] Al-Maskari F. (2010). Lifestyle Diseases: An Economic Burden on the Health Services. UN Chronicle. Available from: https://www.un.org/en/chronicle/article/lifestyle-diseaseseconomicburden-health-services.

[ref6] Amlung M. Mac K. J. (2014). Understanding the effects of stress and alcohol cues on motivation for alcohol via behavioral economics. Alcohol. Clin. Exp. Res. 38, 1780–1789. doi: 10.1111/acer.12423, PMID: 24890323 PMC4049358

[ref7] Andy C. Warnakulasuriya S. Thompson R. P. H. (2003). Neoplasia of the tongue in a patient with Crohn's disease treated with azathioprine: case report. Eur. J. Gastroenterol. Hepatol. 15, 185–187. doi: 10.1097/00042737-200302000-00013 12560764

[ref8] Bagnardi V. Rota M. Botteri E. Tramacere I. Islami F. Fedirko V.. (2015). Alcohol consumption and site-specific cancer risk: a comprehensive dose-response meta-analysis. Br. J. Cancer 112, 580–593. doi: 10.1038/bjc.2014.579, PMID: 25422909 PMC4453639

[ref9] Boileau I. Assaad J. M. Pihl R. O. Benkelfat C. Leyton M. Diksic M.. (2003). Alcohol promotes dopamine release in the human nucleus Accumbens. Synapse 49, 226–231. doi: 10.1002/syn.10226, PMID: 12827641

[ref10] Bryant J. Bonevski B. Paul C. O'Brien J. Oakes W. (2011). Developing cessation interventions for the social and community service setting: a qualitative study of barriers to quitting among disadvantaged Australian smokers. BMC Public Health 11:493. doi: 10.1186/1471-2458-11-493, PMID: 21699730 PMC3135539

[ref11] Carim-Todd L. Mitchell S. H. Oken B. S. (2016). Impulsivity and stress response in nondependent smokers (tobacco chippers) in comparison to heavy smokers and nonsmokers. Nicotine Tob. Res. 18, 547–556. doi: 10.1093/ntr/ntv210, PMID: 26391579 PMC5896825

[ref12] Chang C. P. Siwakoti B. Sapkota A. Gautam D. K. Lee Y. A. Monroe M.. (2020). Tobacco Smoking, chewing habits, alcohol drinking and the risk of head and neck cancer in Nepal. Int. J. Cancer 147, 866–875. doi: 10.1002/ijc.32823, PMID: 31837000 PMC7906484

[ref13] Childs E. de Wit H. (2009). Hormonal, cardiovascular, and subjective responses to acute stress in smokers. Psychopharmacology 203, 1–12. doi: 10.1007/s00213-008-1359-5, PMID: 18936915 PMC2727744

[ref14] Choi Y. J. Myung S. K. Lee J. H. (2018). Light alcohol drinking and risk of cancer: a meta-analysis of cohort studies. Cancer Res. Treat. 50, 474–487. doi: 10.4143/crt.2017.094, PMID: 28546524 PMC5912140

[ref15] Clancy N. Zwar N. Richmond R. (2013). Depression, smoking and smoking cessation: a qualitative study. Fam. Pract. 30, 587–592. doi: 10.1093/fampra/cmt032 23836095

[ref16] Cole G. Tucker L. Friedman G. M. (1990). Relationships among measures of alcohol drinking behavior life-events and perceived stress. Psychol. Rep. 67, 587–591. doi: 10.2466/pr0.1990.67.2.587 2263712

[ref17] Conway D. I. McMahon A. D. Smith K. Black R. Robertson G. Devine J.. (2010). Components of socioeconomic risk associated with head and neck cancer: a population-based case-control study in Scotland. Br. J. Oral Maxillofac. Surg. 48, 11–17. doi: 10.1016/j.bjoms.2009.03.020, PMID: 19481316

[ref18] Cross S. J. Lotfipour S. Leslie F. M. (2017). Mechanisms and genetic factors underlying co-use of nicotine and alcohol or other drugs of abuse. Am. J. Drug Alcohol Abuse 43, 171–185. doi: 10.1080/00952990.2016.1209512, PMID: 27532746 PMC5493323

[ref19] Curtis D. C. Eckhart S. C. Morrow A. C. Sikes L. C. Mridha T. (2020). Demographic and behavioral risk factors for Oral cancer among Florida residents. J. Int. Soc. Prev. Commun. Dent. 10, 255–261. doi: 10.4103/jispcd.JISPCD_39_20, PMID: 32802770 PMC7402261

[ref20] Dal Maso L. Torelli N. Biancotto E. Di Maso M. Gini A. Franchin G.. (2016). Combined effect of tobacco Smoking and alcohol drinking in the risk of head and neck cancers: a re-analysis of case-control studies using Bi-dimensional spline models. Eur. J. Epidemiol. 31, 385–393. doi: 10.1007/s10654-015-0028-3, PMID: 25855002

[ref21] de Wit H. Söderpalm A. H. Nikolayev L. Young E. (2003). Effects of acute social stress on alcohol consumption in healthy subjects. Alcohol. Clin. Exp. Res. 27, 1270–1277. doi: 10.1097/01.Alc.0000081617.37539.D6, PMID: 12966321

[ref22] Doyon W. M. Dong Y. Ostroumov A. Thomas A. M. Zhang T. A. Dani J. A. (2013). Nicotine decreases ethanol-induced dopamine signaling and increases self-administration via stress hormones. Neuron 79, 530–540. doi: 10.1016/j.neuron.2013.06.006, PMID: 23871233 PMC3755901

[ref23] Fallon C. K. Karlawish J. (2019). Is the who definition of health aging well? Frameworks for “health” after three score and ten. Am. J. Public Health 109, 1104–1106. doi: 10.2105/ajph.2019.305177 31268759 PMC6611105

[ref24] Fidler J. A. West R. (2009). Self-perceived Smoking motives and their correlates in a general population sample. Nicotine Tob. Res. 11, 1182–1188. doi: 10.1093/ntr/ntp120, PMID: 19640835

[ref25] Field M. Quigley M. (2009). Mild stress increases attentional bias in social drinkers who drink to cope: a replication and extension. Exp. Clin. Psychopharmacol. 17, 312–319. doi: 10.1037/a0017090, PMID: 19803630

[ref26] Fluharty M. Taylor A. E. Grabski M. Munafò M. R. (2017). The Association of Cigarette Smoking with depression and anxiety: a systematic review. Nicotine Tob. Res. 19, 3–13. doi: 10.1093/ntr/ntw140, PMID: 27199385 PMC5157710

[ref27] Fox H. C. Bergquist K. L. Hong K. I. Sinha R. (2007). Stress-induced and alcohol Cue-induced craving in recently abstinent alcohol-dependent individuals. Alcohol. Clin. Exp. Res. 31, 395–403. doi: 10.1111/j.1530-0277.2006.00320.x, PMID: 17295723

[ref28] Gallo L. C. Roesch S. C. Fortmann A. L. Carnethon M. R. Penedo F. J. Perreira K.. (2014). Associations of chronic stress burden, perceived stress, and traumatic stress with cardiovascular disease prevalence and risk factors in the Hispanic community health study/study of Latinos sociocultural ancillary study. Psychosom. Med. 76, 468–475. doi: 10.1097/psy.0000000000000069, PMID: 24979579 PMC4349387

[ref29] Garey L. Olofsson H. Garza T. Shepherd J. M. Smit T. Zvolensky M. J. (2020). The role of anxiety in Smoking onset, severity, and cessation-related outcomes: a review of recent literature. Curr. Psychiatry Rep. 22:38. doi: 10.1007/s11920-020-01160-5, PMID: 32506166

[ref30] GBD 2019 Risk Factors Collaborators (2020). Global burden of 87 risk factors in 204 countries and territories, 1990-2019: a systematic analysis for the Global Burden of Disease Study 2019. Lancet 396, 1223–1249. doi: 10.1016/s0140-6736(20)30752-2, PMID: 33069327 PMC7566194

[ref31] Goldenberg D. (2002). Maté: a risk factor for oral and oropharyngeal cancer. Oral Oncol. 38, 646–649. doi: 10.1016/s1368-8375(01)00127-0 12167417

[ref32] Goldstein B. Y. Chang S. C. Hashibe M. La Vecchia C. Zhang Z. F. (2010). Alcohol consumption and cancers of the Oral cavity and pharynx from 1988 to 2009: an update. Eur. J. Cancer Prev. 19, 431–465. doi: 10.1097/CEJ.0b013e32833d936d, PMID: 20679896 PMC2954597

[ref33] Graham H. Inskip H. M. Francis B. Harman J. (2006). Pathways of disadvantage and Smoking careers: evidence and policy implications. J. Epidemiol. Commun. Health 60 Suppl 2, 7–12. doi: 10.1136/jech.2005.045583, PMID: 17708005 PMC2491894

[ref34] Hajek P. Taylor T. McRobbie H. (2010). The effect of stopping Smoking on perceived stress levels. Addiction 105, 1466–1471. doi: 10.1111/j.1360-0443.2010.02979.x, PMID: 20528815

[ref35] Harris J. C. (2011). The triumph of Bacchus. Arch. Gen. Psychiatry 68, 8–9. doi: 10.1001/archgenpsychiatry.2010.185, PMID: 21199960

[ref36] Huang Y. Zhao J. Sanghee M. G. Lee G. Zhang J. Bi J.. (2019). Identification of novel genetic variants predisposing to familial oral squamous cell carcinomas. Cell Discov. 5:57. doi: 10.1038/s41421-019-0126-6, PMID: 31798960 PMC6877579

[ref37] IARC. Cancer Today, IARC: Lyon (2020).

[ref97] IARC Working Group on the Evaluation of Carcinogenic Risks to Humans (2004). Tobacco smoke and involuntary smoking. IARC Monogr. Eval. Carcinog. Risks Hum. 83, 1–1438. 15285078 PMC4781536

[ref76] IARC Working Group on the Evaluation of Carcinogenic Risks to Humans (2012). Personal habits and indoor combustions. Volume 100 E. A review of human carcinogens. IARC Monogr. Eval. Carcinog. Risks Hum. 100, 1–538. PMC4781577 23193840

[ref38] Iftikhar A. Islam M. Shepherd S. Jones S. Ellis I. (2021a). Cancer and stress: does it make a difference to the patient when these two challenges collide? Cancers 13:163. doi: 10.3390/cancers13020163, PMID: 33418900 PMC7825104

[ref39] Iftikhar A. Islam M. Shepherd S. Jones S. Ellis I. (2021b). Is Ras the link between Covid-19 and increased stress in head and neck cancer patients? Front. Cell Dev. Biol. 9:714999. Epub 2021/08/03. doi: 10.3389/fcell.2021.714999, PMID: 34336866 PMC8320172

[ref40] International Agency for Research on Cancer. World cancer report: Cancer research for cancer prevention Lyon, France: IARC (2020).

[ref41] Irani S. (2020). New insights into Oral cancer-risk factors and prevention: a review of literature. Int. J. Prev. Med. 11:202. doi: 10.4103/ijpvm.IJPVM_403_18, PMID: 33815726 PMC8000242

[ref42] Kerr S. Watson H. Tolson D. Lough M. Brown M. (2006). Smoking after the age of 65 years: a qualitative exploration of older current and former Smokers' views on Smoking, stopping Smoking, and Smoking cessation resources and services. Health Soc. Care Community 14, 572–582. doi: 10.1111/j.1365-2524.2006.00659.x, PMID: 17059499

[ref43] Keyes K. M. Hatzenbuehler M. L. Grant B. F. Hasin D. S. (2012). Stress and alcohol: epidemiologic evidence. Alcohol res.: curr. rev. 34, 391–400. 10.35946/arcr.v34.4.03 PMC3797525 23584105

[ref44] Khaderi S. A. (2019). Introduction: alcohol and alcoholism. Clin. Liver Dis. 23, 1–10. doi: 10.1016/j.cld.2018.09.009 30454824

[ref45] Kirschbaum C. Pirke K. M. Hellhammer D. H. (1993). The ‘Trier social stress Test’: A tool for investigating psychobiological stress responses in a laboratory setting. Neuropsychobiology 28, 76–81. doi: 10.1159/000119004 8255414

[ref46] Kirschbaum C. Scherer G. Strasburger C. J. (1994). Pituitary and adrenal hormone responses to pharmacological, physical, and psychological stimulation in habitual smokers and nonsmokers. Clin. Investig. 72, 804–810. doi: 10.1007/bf00180552, PMID: 7865987

[ref47] Kumar M. Nanavati R. Modi T. G. Dobariya C. (2016). Oral cancer: etiology and risk factors: a review. J. Cancer Res. Ther. 12, 458–463. doi: 10.4103/0973-1482.186696 27461593

[ref48] Lasser K. Boyd J. W. Woolhandler S. Himmelstein D. U. McCormick D. Bor D. H. (2000). Smoking and mental illness: a population-based prevalence study. JAMA 284, 2606–2610. doi: 10.1001/jama.284.20.2606 11086367

[ref49] Last J. M. (2007). “Definition of health” in A Dictionary of Public Health. ed. Last J. M. (Oxford: Oxford University Press)

[ref50] Lawless M. H. Harrison K. A. Grandits G. A. Eberly L. E. Allen S. S. (2015). Perceived stress and Smoking-related behaviors and symptomatology in male and female smokers. Addict. Behav. 51, 80–83. doi: 10.1016/j.addbeh.2015.07.011, PMID: 26240941 PMC4558262

[ref51] Lawn S. J. Pols R. G. Barber J. G. (2002). Smoking and quitting: a qualitative study with community-living psychiatric clients. Soc. Sci. Med. 54, 93–104. doi: 10.1016/s0277-9536(01)00008-9, PMID: 11820684

[ref52] Lechner W. V. Laurene K. R. Patel S. Anderson M. Grega C. Kenne D. R. (2020). Changes in alcohol use as a function of psychological distress and social support following Covid-19 related university closings. Addict. Behav. 110:106527. Epub 2020/07/18. doi: 10.1016/j.addbeh.2020.106527, PMID: 32679435 PMC7319610

[ref53] Leonardi F. (2018). The definition of health: towards new perspectives. Int. J. Health Serv. 48, 735–748. doi: 10.1177/0020731418782653, PMID: 29902944

[ref54] Lerman C. Audrain J. Orleans C. T. Boyd R. Gold K. Main D.. (1996). Investigation of mechanisms linking depressed mood to nicotine dependence. Addict. Behav. 21, 9–19. doi: 10.1016/0306-4603(95)00032-1, PMID: 8729703

[ref55] LoConte N. K. Brewster A. M. Kaur J. S. Merrill J. K. Alberg A. J. (2018). Alcohol and cancer: a statement of the American Society of Clinical Oncology. J. Clin. Oncol. 36, 83–93. doi: 10.1200/jco.2017.76.1155, PMID: 29112463

[ref56] Lu Y. Sobue T. Kitamura T. Matsuse R. Kitamura Y. Matsuo K.. (2018). Cigarette Smoking, alcohol drinking, and Oral cavity and pharyngeal cancer in the Japanese: a population-based cohort study in Japan. Eur. J. Cancer Prev. 27, 171–179. doi: 10.1097/cej.0000000000000283, PMID: 29324519

[ref57] Magrys S. A. Olmstead M. C. (2015). Acute stress increases voluntary consumption of alcohol in undergraduates. Alcohol Alcohol. 50, 213–218. doi: 10.1093/alcalc/agu101, PMID: 25557606

[ref58] Mann K. Hermann D. Heinz A. (2000). One hundred years of alcoholism: the twentieth century. Alcohol Alcohol. 35, 10–15. Epub 2000/02/24. doi: 10.1093/alcalc/35.1.10, PMID: 10684770

[ref59] Martinez I. (1969). Factors associated with Ccer of the esophagus, mouth, and pharynx in Puerto Rico. J. Natl. Cancer Inst. 42, 1069–1094. PMID: 5793187

[ref60] Mazul A. L. Hartman C. Kramer J. White D. L. Royse K. Raychaudhury S.. (2020). Incidence and survival for oropharynx and non-oropharynx head and neck cancers among veterans living with HIV. Cancer Med. 9, 9326–9335. doi: 10.1002/cam4.3539, PMID: 33094910 PMC7774719

[ref61] McEwen A. West R. McRobbie H. (2008). Motives for Smoking and their correlates in clients attending stop Smoking treatment services. Nicotine Tob. Res. 10, 843–850. doi: 10.1080/14622200802027248, PMID: 18569758

[ref62] McKee S. A. Sinha R. Weinberger A. H. Sofuoglu M. Harrison E. L. Lavery M.. (2011). Stress decreases the ability to resist Smoking and potentiates Smoking intensity and reward. J. Psychopharmacol. 25, 490–502. doi: 10.1177/0269881110376694, PMID: 20817750 PMC3637660

[ref63] Mello F. W. Melo G. Pasetto J. J. Silva C. A. B. Warnakulasuriya S. Rivero E. R. C. (2019). The synergistic effect of tobacco and alcohol consumption on Oral squamous cell carcinoma: a systematic review and meta-analysis. Clin. Oral Investig. 23, 2849–2859. doi: 10.1007/s00784-019-02958-1, PMID: 31111280

[ref64] Milne I. (2011). A Counterblaste to tobacco: king James's anti-Smoking tract of 1616. J. R. Coll. Physicians Edinb. 41:89. doi: 10.4997/JRCPE.2011.118

[ref65] Mishra S. Mishra M. B. (2013). Tobacco: its historical, cultural, Oral, and periodontal health association. J. Int. Soc. Prev. Commun.Dent. 3, 12–18. Epub 2014/01/31. doi: 10.4103/2231-0762.115708, PMID: 24478974 PMC3894096

[ref66] Moreno-López L. A. Esparza-Gómez G. C. González-Navarro A. Cerero-Lapiedra R. González-Hernández M. J. Domínguez-Rojas V. (2000). Risk of Oral cancer associated with tobacco Smoking, alcohol consumption and Oral hygiene: a case-control study in Madrid. Spain. Oral Oncol. 36, 170–174. doi: 10.1016/s1368-8375(99)00084-6, PMID: 10745168

[ref67] Moylan S. Jacka F. N. Pasco J. A. Berk M. (2012). Cigarette Smoking, nicotine dependence and anxiety disorders: a systematic review of population-based Epidemiological Studies. BMC Med. 10:123. doi: 10.1186/1741-7015-10-123, PMID: 23083451 PMC3523047

[ref68] Mullett C. F. (1940). Tobacco as a drug in earlier English medicine. Ann. Med. Hist. 2, 110–123. Epub 1940/03/01. PMID: 33943802 PMC7942597

[ref69] National Center for Chronic Disease Prevention and Health Promotion Office on Smoking and Health (2014). “National Center for Chronic Disease Prevention and Health Promotion Office on Smoking and healthReports of the surgeon general. The health consequences of Smoking—50 years of Progress” in A Report of the Surgeon General (Atlanta (GA): Centers for Disease Control and Prevention (US))

[ref70] Noone M. Dua J. Markham R. (1999). Stress, cognitive factors, and coping resources as predictors of relapse in alcoholics. Addict. Behav. 24, 687–693. doi: 10.1016/s0306-4603(98)00087-2 10574307

[ref71] Ogden G. R. (2018). Alcohol and mouth cancer. Br. Dent. J. 225, 880–883. doi: 10.1038/sj.bdj.2018.921 30412538

[ref72] Okamoto T. Harnett M. T. Morikawa H. (2006). Hyperpolarization-activated cation current (Ih) is an ethanol target in midbrain dopamine neurons of mice. J. Neurophysiol. 95, 619–626. doi: 10.1152/jn.00682.2005, PMID: 16148268 PMC1454360

[ref73] Olsen J. Sabreo S. Fasting U. (1985). Interaction of alcohol and tobacco as risk factors in cancer of the laryngeal region. J. Epidemiol. Commun. Health 39, 165–168. doi: 10.1136/jech.39.2.165, PMID: 4009100 PMC1052426

[ref74] Olshan A. F. Weissler M. C. Watson M. A. Bell D. A. (2001). Risk of head and neck cancer and the alcohol dehydrogenase 3 genotype. Carcinogenesis 22, 57–61. doi: 10.1093/carcin/22.1.57 11159741

[ref75] Park J. O. Nam I. C. Kim C. S. Park S. J. Lee D. H. Kim H. B.. (2022). Sex differences in the prevalence of head and neck cancers: a 10-year follow-up study of 10 million healthy people. Cancers 14:2521. doi: 10.3390/cancers14102521, PMID: 35626129 PMC9139445

[ref77] Petti S. (2009). Lifestyle risk factors for Oral cancer. Oral Oncol. 45, 340–350. doi: 10.1016/j.oraloncology.2008.05.018 18674956

[ref78] Pollay R. W. (2000). Targeting youth and concerned smokers: evidence from Canadian tobacco industry documents. Tob. Control. 9, 136–147. doi: 10.1136/tc.9.2.136, PMID: 10841849 PMC1748318

[ref79] Pomerleau O. F. Pomerleau C. S. (1990). Cortisol response to a psychological stressor and/or nicotine. Pharmacol. Biochem. Behav. 36, 211–213. doi: 10.1016/0091-3057(90)90153-9 2349265

[ref80] Porter S. Gueiros L. A. Leão J. C. Fedele S. (2018). Risk factors and Etiopathogenesis of potentially premalignant Oral epithelial lesions. Oral Surg. Oral Med. Oral Pathol. Oral Radiol. 125, 603–611. doi: 10.1016/j.oooo.2018.03.008, PMID: 29891084

[ref81] Praud D. Rota M. Rehm J. Shield K. Zatoński W. Hashibe M.. (2016). Cancer incidence and mortality attributable to alcohol consumption. Int. J. Cancer 138, 1380–1387. doi: 10.1002/ijc.29890 26455822

[ref82] Proctor R. N. (2004). The global smoking epidemic: a history and status report. Clin. Lung Cancer 5, 371–376. doi: 10.3816/CLC.2004.n.016, PMID: 15217537

[ref83] Public Health England (2018). *Chapter 6: wider Determinants of Health*. London: Public Health England. Available from Chapter 6: wider determinants of health - GOV.UK (www.gov.uk)

[ref84] Raffetti E. Landgren A. J. Andersson F. Donato F. Lavebratt C. Forsell Y.. (2021). Cortisol concentration as predictor of tobacco initiation in adolescents: results from a population-based Swedish cohort. J. Adolesc. Health 68, 758–764. doi: 10.1016/j.jadohealth.2020.08.012, PMID: 33039272

[ref85] Rao U. Hammen C. L. London E. D. Poland R. E. (2009). Contribution of hypothalamic-pituitary-adrenal activity and environmental stress to vulnerability for Smoking in adolescents. Neuropsychopharmacology 34, 2721–2732. doi: 10.1038/npp.2009.112, PMID: 19693006 PMC2784160

[ref86] Reeve M. (2016). Special needs, cheerful habits: Smoking and the great war in Britain, 1914-18. Cultural & Social History 13, 483–501. doi: 10.1080/14780038.2016.1237409

[ref87] Ritz E. Orth S. R. (2000). The cultural history of Smoking. Contrib. Nephrol. 130, 1–10. doi: 10.1159/000060053 10892545

[ref88] Russell M. Cooper M. L. Frone M. R. Peirce R. S. (1999). A longitudinal study of stress, alcohol, and blood pressure in community-based samples of blacks and non-blacks. Alcohol Res. Health 23, 299–306. PMID: 10890827 PMC6760380

[ref89] Sabatini M. E. Chiocca S. (2020). Human papillomavirus as a driver of head and neck cancers. Br. J. Cancer 122, 306–314. doi: 10.1038/s41416-019-0602-7, PMID: 31708575 PMC7000688

[ref90] Shield K. Manthey J. Rylett M. Probst C. Wettlaufer A. Parry C. D. H.. (2020). National, regional, and global burdens of disease from 2000 to 2016 attributable to alcohol use: a comparative risk assessment study. Lancet Public Health 5, e51–e61. doi: 10.1016/s2468-2667(19)30231-2, PMID: 31910980

[ref91] Slopen N. Kontos E. Z. Ryff C. D. Ayanian J. Z. Albert M. A. Williams D. R. (2013). Psychosocial stress and cigarette Smoking persistence, cessation, and relapse over 9-10 years: A prospective study of middle-aged adults in the United States. Cancer Causes Control 24, 1849–1863. doi: 10.1007/s10552-013-0262-5, PMID: 23860953 PMC3776130

[ref92] Smith E. A. Malone R. E. (2009). “Everywhere the soldier will be”: wartime tobacco promotion in the us military. Am. J. Public Health 99, 1595–1602. doi: 10.2105/ajph.2008.152983, PMID: 19608945 PMC2724442

[ref93] Speight P. M. Khurram S. A. Kujan O. (2018). Oral potentially malignant disorders: risk of progression to malignancy. Oral Surg. Oral Med. Oral Pathol. Oral Radiol. 125, 612–627. doi: 10.1016/j.oooo.2017.12.011 29396319

[ref94] Stephens M. A. Wand G. (2012). Stress and the Hpa Axis: role of glucocorticoids in alcohol dependence. Alcohol Res. 34, 468–483. 23584113 10.35946/arcr.v34.4.11 PMC3860380

[ref95] Stewart G. G. (1967). A history of the medicinal use of tobacco 1492-1860. Med. Hist. 11, 228–268. Epub 1967/07/01. doi: 10.1017/s0025727300012333, PMID: 4864420 PMC1033728

[ref96] Su C. C. Tsai K. Y. Hsu Y. Y. Lin Y. Y. Lian I. (2010). Chronic exposure to heavy metals and risk of oral cancer in Taiwanese males. Oral Oncol. 46, 586–590. doi: 10.1016/j.oraloncology.2010.05.001, PMID: 20619722

[ref98] The Germicidal Properties of Tobacco Smoke (1913). Lancet 181:406. doi: 10.1016/S0140-6736(01)20259-1

[ref99] Thomas S. E. Bacon A. K. Randall P. K. Brady K. T. See R. E. (2011). An acute psychosocial stressor increases drinking in non-treatment-seeking alcoholics. Psychopharmacology 218, 19–28. doi: 10.1007/s00213-010-2163-6, PMID: 21274703 PMC3115478

[ref100] Thompson K. Dutton D. J. Mac Nabb K. Liu T. Blades S. Asbridge M. (2021). Changes in alcohol consumption during the Covid-19 pandemic: exploring gender differences and the role of emotional distress. Health Promot. Chronic Dis. Prev. Can. 41, 254–263. doi: 10.24095/hpcdp.41.9.02, PMID: 34164972 PMC8565493

[ref101] Thompson B. Thompson L. A. Thompson J. Fredickson C. Bishop S. (2003). Heavy smokers: a qualitative analysis of attitudes and beliefs concerning cessation and continued Smoking. Nicotine Tob. Res. 5, 923–933. doi: 10.1080/14622200310001615277, PMID: 14668076

[ref102] Tobias M. Templeton R. Collings S. (2008). How much do mental disorders contribute to New Zealand's tobacco epidemic? Tob. Control. 17, 347–350. doi: 10.1136/tc.2008.026005, PMID: 18669558

[ref103] Tsourtos G. Ward P. R. Muller R. Lawn S. Winefield A. H. Hersh D.. (2011). The importance of resilience and stress to maintaining Smoking abstinence and cessation: a qualitative study in Australia with people diagnosed with depression. Health Soc. Care Community 19, 299–306. doi: 10.1111/j.1365-2524.2010.00973.x, PMID: 21138494

[ref104] Vendruscolo L. F. Barbier E. Schlosburg J. E. Misra K. K. Whitfield T. W. Logrip M. L.. (2012). Corticosteroid-dependent plasticity mediates compulsive alcohol drinking in rats. J. Neurosci. 32, 7563–7571. doi: 10.1523/jneurosci.0069-12.2012, PMID: 22649234 PMC3375621

[ref105] Viner R. (1999). Putting stress in life: Hans Selye and the making of stress theory. Soc. Stud. Sci. 29, 391–410. doi: 10.1177/030631299029003003

[ref106] Waltman C. Blevins L. S. Boyd G. Wand G. S. (1993). The effects of mild ethanol intoxication on the hypothalamic-pituitary-adrenal Axis in nonalcoholic men. J. Clin. Endocrinol. Metab. 77, 518–522. doi: 10.1210/jcem.77.2.8393888, PMID: 8393888

[ref107] Warnakulasuriya S. (2009). Causes of Oral cancer: an appraisal of controversies. Br. Dent. J. 207, 471–475. doi: 10.1038/sj.bdj.2009.1009, PMID: 19946320

[ref108] Wilsnack S. C. Klassen A. D. Schur B. E. Wilsnack R. W. (1991). Predicting onset and chronicity of Women's problem drinking: a five-year longitudinal analysis. Am. J. Public Health 81, 305–318. doi: 10.2105/ajph.81.3.305, PMID: 1994739 PMC1405008

[ref109] World Health Organization (1986). Ottawa Charter for Health Promotion, 1986 Ottawa. Canada: World Health Organization.

[ref110] World Health Organization (2021). Who Global report on trends in Prevalence of Tobacco Use 2000–2025. Geneva: World Health Organization.

[ref111] World Health Organization (2022). *Preventing Cancer*. Available from https://www.who.int/activities/preventing-cancer.

[ref112] Wu L. T. Anthony J. C. (1999). Tobacco Smoking and depressed mood in late childhood and early adolescence. Am. J. Public Health 89, 1837–1840. doi: 10.2105/ajph.89.12.1837, PMID: 10589312 PMC1509011

[ref113] Wynder E. L. Bross I. J. (1957). Aetiological factors in mouth cancer; an approach to its prevention. Br. Med. J. 1, 1137–1143. doi: 10.1136/bmj.1.5028.1137, PMID: 13426561 PMC1973476

[ref114] Zaman R. Hankir A. Jemni M. (2019). Lifestyle factors and mental health. Psychiatr. Danub. 31, 217–220. 31488729

